# Author Correction: Between hope and reality: treatment of genetic diseases through nucleic acid-based drugs

**DOI:** 10.1038/s42003-025-08481-2

**Published:** 2025-07-10

**Authors:** Virginie Baylot, Thi Khanh Le, David Taïeb, Palma Rocchi, Laurence Colleaux

**Affiliations:** Aix Marseille Univ, CNRS, CINAM, ERL INSERM U 1326, CERIMED, Marseille, France

**Keywords:** Drug development, Genetics research

Correction to: *Communications Biology* 10.1038/s42003-024-06121-9, published online 23 April 2024

In this article, the ‘Competing Interest’ statement was incorrectly given as ‘The authors declare no competing interests’ but should have been ‘P. Rocchi, D. Taieb and L. Colleaux are cofounders of SilonTx (https://silontx.com), a biotech company started in April 2024 focusing on precision medicine and nucleic acid therapeutics. The other authors declare no competing interests’. The original article has been corrected.

