# Supplementary material for: Between hope and reality: treatment of genetic diseases through nucleic acid-based drugs
Source: Commun Biol. 2024 Apr 23;7:489. doi: 10.1038/s42003-024-06121-9 (PMC11039704; doi:10.1038/s42003-024-06121-9)
Supplement: Supplementary file 2 — Supplementary Information [file 42003_2024_6121_MOESM2_ESM.pdf]

Supplementary Information:

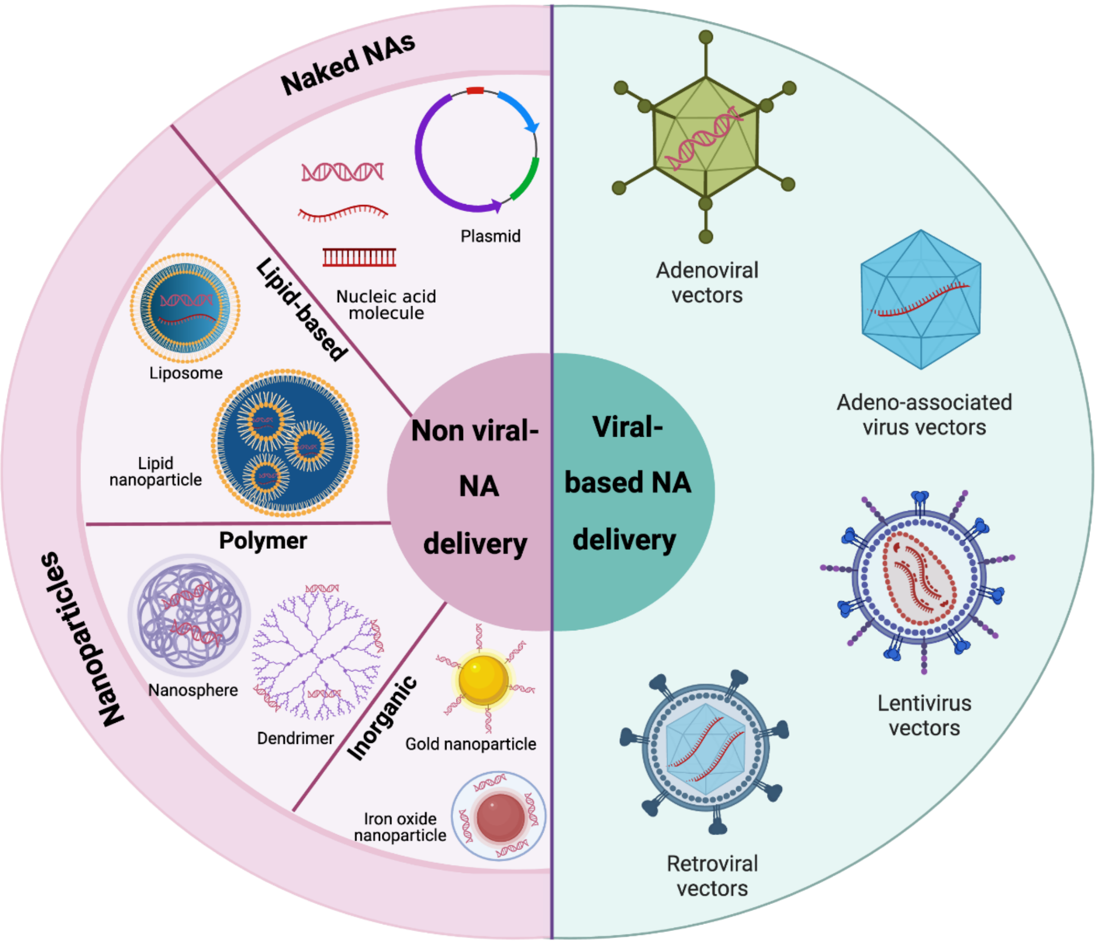

Supplementary Figure 1: Main NA-based therapeutics delivery systems. Delivery systems for NA drugs can be classified into two main categories: viral-based vectors and non-viral delivery systems. Among the viral vectors, adenoviral vectors (Ads), adeno-associated viral vectors (AAVs), lentiviral vectors, and retroviral vectors are the most frequently used due to their high efficiency and safety profile. Adenoviral vectors are particularly effective for gene transfer in non-dividing cells, while adeno-associated viral vectors have low immunogenicity and are capable of long-term transgene expression. Lentiviral vectors can integrate their genetic material into the host genome, allowing for stable and long-term gene expression. Retroviral vectors, on the other hand, are useful for gene therapy applications that require targeting of dividing cells. NA molecules can be given without delivery aides, which can be more convenient and less expensive than using aides. However, this may reduce their effectiveness due to degradation and clearance by the body. It is important to carefully weigh the potential risks and benefits and consult a healthcare professional or expert before using this method. Nanoparticles have emerged as a promising delivery system for a variety of drugs. The delivery of drugs using nanoparticles is usually achieved using lipid-based NPs such as liposome and lipid nanoparticles, polymeric-based NPs like nanosphere and dendrimer, and inorganic NPs such as gold nanoparticle and iron oxide nanoparticle. Each of these nanoparticle types has unique advantages and disadvantages. For example, lipid-based NPs are biocompatible, biodegradable, and can encapsulate both hydrophilic and hydrophobic drugs. On the other hand, polymeric-

based NPs offer a high drug loading capacity and can be engineered to target specific cells or tissues. Inorganic NPs, such as gold nanoparticles, can be used for imaging and drug delivery due to their unique optical and electronic properties. Created with BioRender.com (Agreement number : ZQ26ML0TTI).

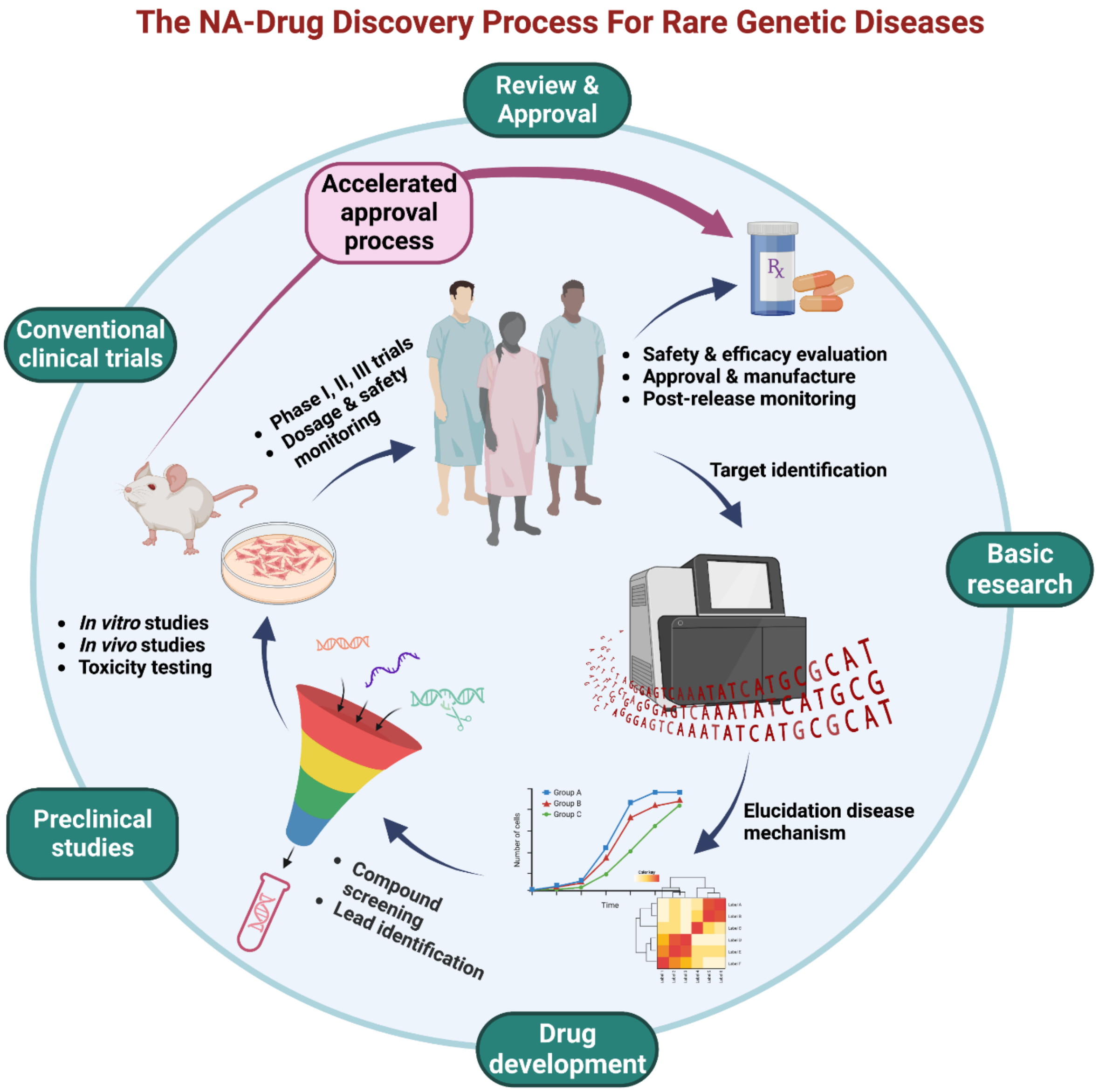

Supplementary Figure 2: The process of drug development is a complex one that involves various stages. At the outset, researchers carry out fundamental research to identify therapeutic targets in patients using a range of advanced technologies. Once these targets are identified, researchers delve deeper into the mechanism behind the disorder to gain a better understanding of how to develop a drug. During the drug development stage, compounds are designed and screened to identify the most promising candidates. These candidates are then tested in preclinical studies using in vitro or in vivo models to gather information on any potential toxicities. This is a crucial step in ensuring the safety of the drug for human use. After the preclinical studies, clinical trials are conducted on the most promising candidate compounds. Clinical trials are done in humans to determine the safety and efficacy of the drug before it can be submitted for authorization. This phase can take several years and involves multiple stages of testing to ensure that the drug is safe and effective.

On the other hand, it is essential to expedite drug development for rare and orphan diseases to offer treatments to patients with limited options. Traditional clinical trials and reviews can take several years and may not guarantee success. These programs allow drug candidates to be administered directly to patients without undergoing traditional clinical trials and reviews. This approach can significantly reduce the time it takes to develop and test new drugs, while also providing patients with much-needed treatments sooner. Furthermore, these programs can also help to overcome some of the challenges associated with traditional clinical trials and reviews. For example, in rare and orphan diseases, it can be challenging to find enough eligible patients to participate in a trial. However, by administering drugs directly to patients, these programs can bypass some of these challenges and provide treatments to those who need them most. Created with BioRender.com (Agreement number : FS26ML279G).

**Drug Name**	**NCT Identifier**	**Phase**	**Delivery strategy**	**Targeted sequence**	**Disease**	**Company**
EDIT-101	NCT03872479	I/II	*In vivo* gene editing delivered by AAV	CEP290 mutation	LCA10	Editas Medicine, Inc
NTLA-2001	NCT04601051	I/II	*In vivo* gene editing delivered by LNPs	Transthyretin (TTR)	ATTR	Intellia therapeutics
NTLA-2002	NCT05120830	I/II	In vivo gene editing delivered by LNPs	kallikrein B1 (KLKB1)	HAE	Intellia therapeutics
VERVE-101	NCT05398029	Ib	In vivo gene editing delivered by LNPs	PCSK9	HeFH, ASCVD	Verve Therapeutics

Supplementary Table 1: Current clinical trials of CRISP-Cas9 therapeutics

	**Advantages**	**Disadvantages**	**References**
**Adenovirus (Ads)**	✓ Large payload capacity (up to 36Kb)<br>✓ High transduction efficiency<br>✓ The viral genome remains in epichromosomal (not integrate into the host chromatins)<br>✓ Broad potrism (both diving cells and quiescent cells and various cell types)	✓ Pre-existing viral immune response<br>✓ Strong immunogenicity against the viral capsid proteins and the transgenic proteins	[1]<br>[2]
**Adeno-Associated Virus (AAV)**	✓ Non-pathogenic virus, simple genome, and structure<br>✓ Low immunogenicity (relatively safe)<br>✓ Bring long-term and effective expression for the therapeutic genes in broad cell types	✓ Small packing capacity (< 5 Kb)<br>✓ Adaptive immuno-stimulation induced by the viral capsid proteins<br>✓ Possibility of oncogenic incorporation of AAV genome into host chromatins<br>✓ High cost	[169,173,3–5]
**Lentivirus**	✓ Integrating vector: long-term transgene expression ability for *ex-vivo* therapy<br>✓ Transduction into both dividing cells and quiescent cells	✓ High risk of mutagenesis insertion: generation of chimeric gene fusions made up of the proviral and host sequences; induction of splicing and	[169,6–9]

	✓ Very low immunogenicity	production of aberrant transcripts	
**Herpes Simplex Virus (HSV)**	✓ Episomal delivery<br>✓ Broad tissue tropism,<br>✓ High transduction efficiency,<br>✓ Large transgene capacity<br>✓ Ability to resist immune clearance *via* the inhibition of innate and adaptive anti-viral immunity	✓ So far, only been validated clinically in dermatology<br>✓ Strong cytopathogenicity	[10–12]

Supplementary Table 2: Pros and Cons of the most commonly used viral-based vectors: Ads, AAVs, lentivirus and HSV

Supplementary references

1. Lee, C. S. *et al.* Adenovirus-mediated gene delivery: Potential applications for gene and cell-based therapies in the new era of personalized medicine. *Genes Dis.* **4**, 43–63 (2017).
2. Crystal, R. G. Adenovirus: The First Effective In Vivo Gene Delivery Vector. *Hum. Gene Ther.* **25**, 3–11 (2014).
3. Pupo, A. *et al.* AAV vectors: The Rubik's cube of human gene therapy. *Mol. Ther.* **30**, 3515–3541 (2022).
4. Mays, L. E. & Wilson, J. M. The Complex and Evolving Story of T cell Activation to AAV Vector-encoded Transgene Products. *Mol. Ther.* **19**, 16–27 (2011).
5. Rogers, G. L. *et al.* Unique Roles of TLR9- and MyD88-Dependent and -Independent Pathways in Adaptive Immune Responses to AAV-Mediated Gene Transfer. *J. Innate Immun.* **7**, 302–314 (2015).
6. Almarza, D. *et al.* Risk assessment in skin gene therapy: viral–cellular fusion transcripts generated by proviral transcriptional read-through in keratinocytes transduced with self-inactivating lentiviral vectors. *Gene Ther.* **18**, 674–681 (2011).
7. Moiani, A. *et al.* Lentiviral vector integration in the human genome induces alternative splicing and generates aberrant transcripts. *J. Clin. Invest.* **122**, 1653–1666 (2012).
8. Modlich, U. *et al.* Insertional Transformation of Hematopoietic Cells by Self-inactivating Lentiviral and Gammaretroviral Vectors. *Mol. Ther.* **17**, 1919–1928 (2009).
9. Cesana, D. *et al.* Uncovering and Dissecting the Genotoxicity of Self-inactivating Lentiviral Vectors In Vivo. *Mol. Ther.* **22**, 774–785 (2014).
10. Mody, P. H., Pathak, S., Hanson, L. K. & Spencer, J. V. Herpes Simplex Virus: A Versatile Tool for Insights Into Evolution, Gene Delivery, and Tumor Immunotherapy. *Virol. Res. Treat.* **11**, 1178122X20913274 (2020).
11. Verlengia, G. *et al.* Engineered HSV vector achieves safe long-term transgene expression in the central nervous system. *Sci. Rep.* **7**, 1507 (2017).
12. Ingusci, S., Verlengia, G., Soukupova, M., Zucchini, S. & Simonato, M. Gene Therapy Tools for Brain Diseases. *Front. Pharmacol.* **10**, (2019).
